# Berberine confers neuroprotection in coping with focal cerebral ischemia by targeting inflammatory cytokines

**DOI:** 10.1016/j.jchemneu.2017.04.008

**Published:** 2018-01

**Authors:** Solmaz Nasseri Maleki, Nahid Aboutaleb, Faramarz Souri

**Affiliations:** Physiology Research Center and Department of Physiology, Faculty of Medicine, Iran University of Medical Sciences, Tehran, Iran

**Keywords:** TTC, 23,5-triphenyltetrazolium chloride, MCAO, middle cerebral artery occlusion, p-AKT, phosphorylated Protein kinase B, pGSK, phosphorylated Glycogen synthase kinase, pCREB, phosphorylated cAMP response element-binding protein, NF-κB, nuclear factor-κB, PI3K, phosphoinositide 3-kinase, AMPK, AMP-activated protein kinase, JAK2, Janus Kinase 2, STAT3, signal transducer and activator of transcription 3, TNFα, tumor necrosis factor alpha, MAPK or MAP kinase, A mitogen-activated protein kinase, PPARγ, peroxisome proliferator-activated receptor-γ, MMP-9, matrix metallopeptidase 9, JNKs, c-Jun N-terminal kinases, IL-1β, interleukin-1β, iNOS, inducible nitric oxide synthase, COX-2, prostaglandin-endoperoxide synthase 2, IL-10, interleukin 10, Berberine, Inflammatory cytokines, MCAO model, Neuroprotection

## Abstract

**Scope:**

Existing research indicates that anti-inflammatory and antioxidant properties of berberine play major roles in coping with oxidative stress in neurodegenerative diseases, but it is not known if this isoquinoline alkaloid affects inflammatory cytokines such as interleukin 10 in focal cerebral ischemia.

**Methods and results:**

Male Wistar rats (10 weeks old) were treated with 40 mg/kg concentration of berberine 1 h after focal cerebral ischemia and the anti-inflammatory properties of berberine were evaluated by immunohistochemical analysis, water content measure and behavioral tests. Evaluation of infarct volume was performed by TTC staining. Immunohistochemistry and behavioral assessment indicated recovery in treatment group compared to only ischemia group. The infarct volume decreased in treatment group compared to ischemia group. Berberine administration significantly decreased brain edema and contributed to the restoration of motor function. Moreover, berberine potently contributed to neuroprotection in motor area through downregulation of pro-inflammatory cytokines and upregulation of anti-inflammatory cytokines.

**Conclusions:**

These findings confirm the validity of berberine as a potent anti-inflammatory agent in treatment of ischemic stroke.

## Highlights

• Berberine reduces brain edema and infarct volume through regulation of inflammatory responses in focal cerebral ischemia.
• Berberine increases the expression of anti-inflammatory cytokines after ischemic stroke.
• Berberine contributes to recovery of motor function after focal cerebral ischemia.

## 1 Introduction

Ischemic stroke is the second leading cause of death which poses an ever-increasing challenge around the world, and is associated with sociocultural and lifestyle changes (Feigin et al., 2014).

Pharmacologic studies have introduced many novel therapeutic agents that have enhanced the recovery of stroke and other diseases (Liu et al., 2013; Jazayeri et al., 2016a, Jazayeri et al., 2016b). Several treatment options such as thrombolysis have been identified for stroke. Adverse side effects of these approaches make them limited. Therefore, new therapeutic agents or approaches are required to increase life expectancy in patients with stroke. During stroke and neurodegenerative diseases, different signaling pathways are involved in cell fate (Mehrjerdi et al., 2013; Ajami et al., 2016; Pazoki-Toroudi et al., 2016). Targeting these signaling pathways by therapeutic agent postpones neuronal death in the brain after cerebral ischemia. Berberine is a yellow plant isoquinoline alkaloid isolated from herb medicine Coptidis Rhizom, Hydrastis canadensis, and berberis that plays an important role in neuroprotection against pro-inflammatory responses and escape from apoptotic signals (Refaat et al., 2013). A large number of pharmacological and biological properties of berberine have been identified including anti carcinogenic (Lu et al., 2016), anti-inflammatory (Guo et al., 2016; Woo et al., 2016), anti-microbial (Wen et al., 2016), antihypertensive (Saki et al., 2016) and cholesterol lowering effects (Wang et al., 2014).

Existing researches have shown that berberine can ameliorate ischemia/reperfusion injury through several mechanisms:

One, berberine can up-regulate pAkt, pGSK and pCREB, and down-regulate NF-κB that resulted in cell survival in the acute phase of cerebral ischemia (Zhang et al., 2012; Yu et al., 2015). Two, it can activate Akt and PI3K p55γ promoter which in turn reduces pro-apoptotic factors such as Bad and caspase-3 (Hu et al., 2012; Yu et al., 2015). Three, the anti-apoptotic effect of berberine is due to inhibition of AMPK pathway (Chen et al., 2016). Four, berberine inhibits apoptosis through downregulation of Bax, caspase 3, caspase 8, caspase 9 and subsequently induces autophagy in focal cerebral ischemia (Zhang et al., 2016). Five, it activates JAK2/STAT3 signaling and inhibits endoplasmic reticulum stress (Zhao et al., 2016). Likewise, current studies have shown anti-inflammatory effects of berberine against transient global cerebral ischemia in animal models (Singh and Chopra, 2013). Berberine may confer neuroprotection against ischemia via modulation of potassium currents in CA1 pyramidal neurons of hippocampus in vitro model (Wang et al., 2004). Moreover, berberine helps to inhibit N-methyl-D-aspartate receptor 1 immunoactivity which in turn resulting in cell survival in CA1 hippocampus region after cerebral ischemia (Ye et al., 2009). Herein, we evaluate the effects of berberine on inflammation and inflammatory cytokines. Berberine might be used as a potential therapeutic agent to reduce inflammation and neuronal demise after ischemic stroke.

## 2 Materials and methods

### 2.1 Chemicals

Berberine) B3251 SIGMA) chloral hydrate (C8383 SIGMA), paraformaldehyde (158127 SIGMA) and 2,3,5-triphenyltetrazolium chloride (T8877 SIGMA(were obtained from Sigma Chemical Co. Silicon rubber-coated monofilament for MCAO model was purchased from Doccol company (Philadelphia, PA, USA). Anti-IL10 antibody (ab34843), Anti-IL1 beta antibody (ab2105) and anti-TNFα (ab9635) were purchased from Abcam Company (Cambridge, UK). Goat anti-rabbit IgG-FITC Secondary Antibody was obtained from Santa Cruz Biotechnology's company.

### 2.2 Animals and ethical statement

Ten-week-old male Wistar rats (200–250 g) were purchased from animal laboratory of Iran University of Medical Sciences, and all experimental tests and the procedures were confirmed by the Institutional Animal Ethical Committee of Iran University of Medical Sciences. Rats were maintained under standard laboratory conditions (in a controlled temperature 25 ± 1 °C, with a 12-h dark: 12-h light cycle, 60% humidity, sterile water and food). The animals were randomly assigned into four groups: a control group of healthy rats (*n* = 20), sham-operated control group (sham, *n* = 20), only ischemia + saline (MCAO, *n* = 20), and treatment group (MCAO + Berberine, *n* = 20).

### 2.3 MCAO model

The animals were anesthetized with 10% chloral hydrate (400 mg/kg body weight) by intraperitoneal injection. To make transit focal ischemia, MCAO model was used as described previously. In sum, a midline neck incision was made to isolate the right common carotid artery, external carotid artery and internal carotid artery. After occlusion of the common carotid artery by micro-clip, external carotid artery was clamped and a monofilament nylon suture was inserted from the right common carotid artery to the internal carotid artery by stumping external carotid artery and gradually moved until mild resistance was felt, confirming the middle cerebral artery occlusion. After 45-min ischemia, the monofilament was removed to allow recirculation of cerebral blood flow for 24 h and the skin incision was closed by surgical clips. During the whole surgery, body temperature of animals were monitored and maintained at 37 °C via measurement by a rectal probe. The same procedure was used for sham-operated control rats except filament insertion.

### 2.4 Neurobehavioral evaluation

Neurobehavioral assessments were performed 24 h after reperfusion by an observer blinded to the treatment groups. A six-point scale by Longa et al. was used to perform neurobehavioral assessments (Longa et al., 1989). 0, no deficits; 1, failure in fully extending the contralateral forelimb; 2, failure to extend the contralateral forelimb; 3, circling to the contralateral side; 4, falling to the left; 5, no spontaneously walking and exhibition of a depressed level of consciousness.

### 2.5 Anesthesia and tissue collection

To examine histochemical analysis, the animals were finally anesthetized at the end of behavioral assessments and after decapitating animals, their brains were removed. Cortex regions of brain were dissected and maintained at –80 °C prior to immunohistochemical analysis.

### 2.6 Infarct volume determination

After the behavioral assessments, the animals were re-anesthetized and decapitated. The brains were quickly cooled in iced saline for 10 min, and then were cut by a brain matrix to several 2 mm thick coronal sections. The sections were incubated in TTC at 37 °C for 20 min, and then were fixed in 10% buffered formalin solution. A digital camera was used to take photograph of the slices. Infarct volume was identified as unstained regions and was calculated through an image analysis software.

### 2.7 Determination of brain water content

Brain water content was evaluated via wet/dry method in different groups (*n* = 4 for each group). In summary, rats were decapitated under an over dose mixture of ketamine and xylazine after 24 h reperfusion. The brains were divided into ischemic and contralateral hemispheres. Ischemic hemisphere was weighed (wet weight) then placed in an oven at 110 °C f for 24 h and again was weighed (dry weight). Brain water content was determined via (wet weight − dry weight)/wet weight * 100%.

### 2.8 Immunohistochemistry

Goat serum (10%) was used to block 5-micrometer sections from paraffin embedded brain tissue. Slices were incubated with primary antibody (1:100) (TNFα antibody, IL-1β and IL-10) at 1 μg/ml in 1% BSA/PBS for 1 h, followed by incubation with specific horseradish peroxidase-conjugated secondary antibody (1:150), each for 30 min. The sections were visualized with a microscope (Olympus, Japan). Quantification of immunohistochemical assay was based on fluorescence intensity.

### 2.9 Statistical analysis

Values were expressed as means ± SD. Analysis was performed using Prism Software, version 5. Statistical difference between two groups was determined by two-tailed Student's *t* tests. Comparison of three or more groups was performed using One-way analysis of variance followed by LSD test for post hoc analysis. Values were considered statistically significant in *p* < 0.05.

## 3 Results and discussion

### 3.1 Neurological outcomes and infarct volume

**Fig. 1:**
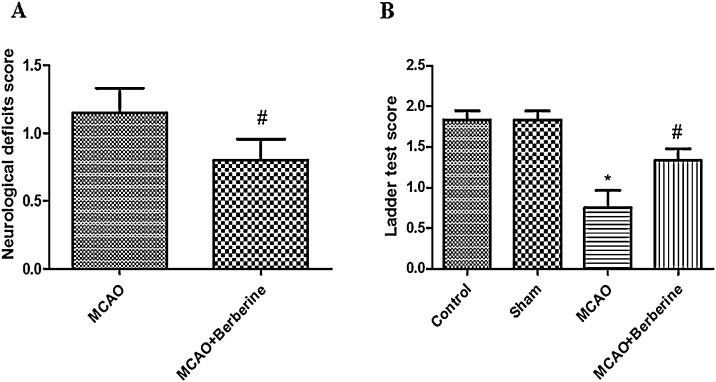
Berberine treatment improves neurological outcomes. (A) Post-treatment by berberine significantly decreases neurological deficits score (^#^*p* < 0.01 compared with ischemia group). (B) Neurological deficits were increased in ischemia group compared to control and sham groups (*p < 0.001 compared to control and sham group). Ladder test displays recovery of treatment group compared to ischemia group (^#^*p* < 0.05 compared to ischemia group).

Previous studies have shown that following transient focal cerebral ischemia oxidative stress and overexpression of pro-inflammatory cytokines cause neurologic deficits, brain edema and neuronal death (Liu et al., 2015a; Yao et al., 2015; Shinohara et al., 2016). To evaluate neuroprotective effect of berberine, we first examined neurobehavioral function through behavioral assessment. Tianpei and coworker reported that berberine can ameliorate neurologic deficits and infarct volume in global ischemia (Wu et al., 1994). Our results show that there were no significant neurological deficits between control and sham groups. As shown in Fig. 1, significant difference (*p* < 0.01) was observed between the ischemia and treatment groups. Treatment group displayed a significant reduction in neurological deficits score compared to ischemia group (*n* = 8 for each group, *p* < 0.01). Likewise, the results depicted in Fig. 1B by ladder test display following 24 h after reperfusion; .Statistical significant differences were observed between control and ischemia cohorts (*n* = 8 for each group, *p* < 0.001). Berberine administration significantly restores motor function than ischemia group (*p* < 0.05).

It has been identified that berberine can attenuate infarct volume in MCAO animal model (Zhou et al., 2008). To examine infarct volume, the slices were immersed in a solution of 2% TTC, and after 20 min white areas were described as the infarcted brain tissue and red areas were defined as the non-infarcted region (Chen et al., 2015).

**Fig. 2:**
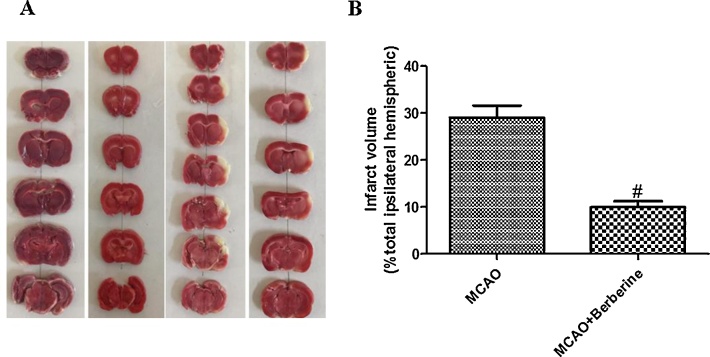
Berberine post-treatment reduced infarction volume in rats. (A) The sections were stained by immersing them in a solution of TTC and non-infarcted region appeared red, whereas the infarcted region appeared white. (B) Berberine administration significantly reduces total infarct volume (^#^*p* < 0.01 compared with ischemia group).

As shown in Fig. 2a and b, no significant differences between control and ischemia group were observed by TTC staining. Berberine post treatment reduced infarction volumes. The infarct volume of the treatment group was 22% lower compared with ischemia group (*n* = 4 for each group, *p* > 0.01).

### 3.2 Brain water content

Brain edema plays an important role in the pathophysiology of neurologic deficits such as stroke and is a common sequel of post-injury inflammation and blood-brain barrier (BBB) breakdown (Manley et al., 2000). Cerebral water balance is hamper in cerebral ischemia (Zhang et al., 2006). There is a close association between inflammation and brain edema. It seems that overexpression of interleukin-1 receptor antagonist can highly attenuate brain edema (Masada et al., 2001). Accumulative evidences have shown that berberine can contribute to normalization of cerebral water balance after brain injury via inhibition of Glia-mediated inflammation (Chen et al., 2014). To evaluate the anti-inflammatory effects of berberine, we measured brain water content in different groups. Following 24 h reperfusion, water content of the brain tissue for the control, sham, ischemia and treatment groups were77.35%, 78.83%, 80.63%, 77.89% respectively. Our results indicated a significant difference between sham and ischemia groups (*p* < 0.01).

**Fig. 3:**
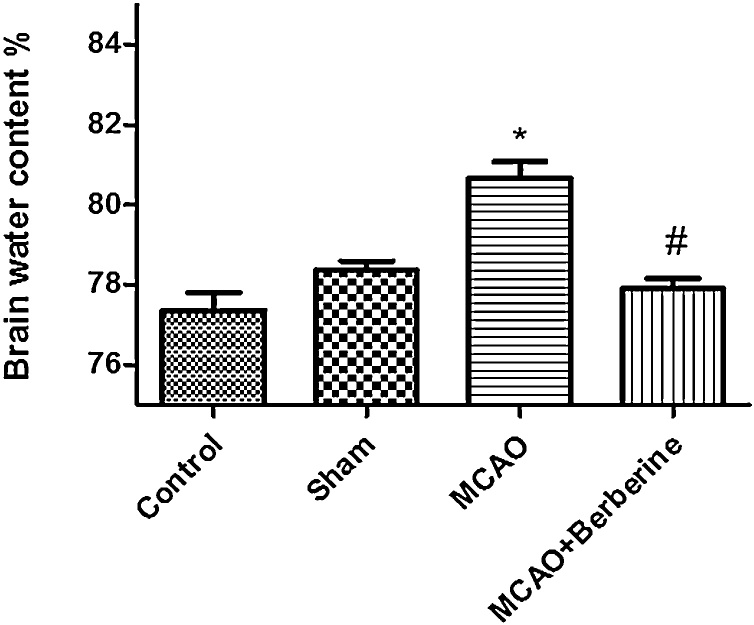
Berberine post-treatment decreased brain edema. Brain water contentsignificantly increased in MCAO compared to control group (*p < 0.01). There was a statistical difference between MCAO and MCAO + Berberine groups, confirming anti-inflammatory effect of Berberine (^#^*p* < 0.001 compared with MCAO group).

Water content of the brain increased significantly in ischemia group compared to control and sham group. Decreased water content observed in treatment group than ischemia group giving clear evidence that berberine alleviates brain edema formation following ischemic stroke (Fig. 3).

### 3.3 Immunohistochemical analysis

Exiting researches have shown that berberine can contribute to neuroprotection in coping with oxidative stress and ischemic stroke through targeting different signaling pathways (Zhang et al., 2012). In addition, this isoquinoline alkaloid can interact with mRNAs that lead to up-regulation of several proteins and escape of proapoptic signals (Chai et al., 2014). Berberine can inhibit proinflammatory responses via AMPK activation in macrophages (Jeong et al., 2009). Recent studies have shown that berberine might downregulate proinflammatory cytokines in a AMPK-independent manner (Woo et al., 2016). Likewise, Berberine can suppress proinflammatory responses through downregulation of proinflammatory cytokines such as TNFα and IL-6 in AcLDL-stimulated macrophages via PPARγ signaling pathway (Chen et al., 2008; Spatuzza et al., 2014). Moreover, Berberine may decrease inflammation by targeting MAPK pathway that in turn suppresses the expression of proinflammatory cytokines (Li et al., 2014). Berberine inhibits cell invasion by supressing TNF-α-induced MMP-9 in human breast cancer (Kim et al., 2008).

**Fig. 4:**
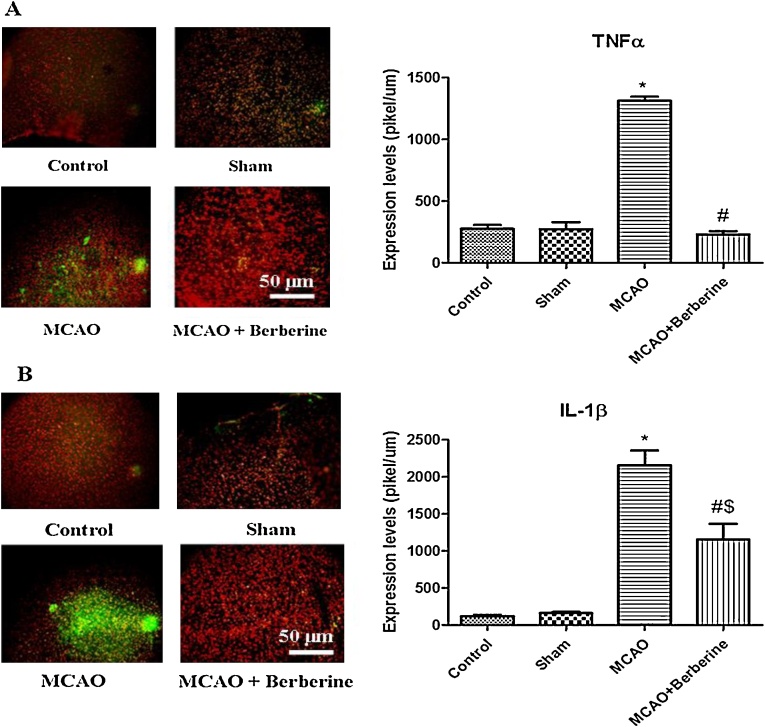
(A) Berberine post-treatment decreased TNFα level. Immunohistochemical analysis results demonstrated that MCAO markedly increased the protein levels of TNFα compared with control and sham groups (*p < 0.001 compared to control and sham groups). TNFα proinflammatory cytokine was significantly decreased compared to ischemia group (^#^*p* < 0.001). (B) MCAO induced a robust increase in IL-1β levels (*p < 0.001 compared to control and sham groups). Berberine post-treatment decreased the level of IL-1β pro-inflammatory cytokine (^#^*p* < 0. 01 compared to MCAO group) (^$^*p* < 0. 01 compared to control and sham groups).

In present work, immunohistochemical studies have shown that TNFα expression was signtificantly increased than control and sham groups (*p* < 0.001). Berberine highly inhibited the expression of TNFα compared to MCAO cohorts (Fig. 4A). Our results displayed berberine administration exhibited a significant anti-inflammatory effect on cerebral ischemia through supression of proinflammatory cytokines.

It has been recognized that berberine can decrease inflammatory agents-induced interleukin-1β and TNFα ensuing inflammation in human lung cells (Lee et al., 2007). Many emerging evidences have shown that berberine contributes to cell survival and inhibition of inflammation by downregulating IL-1β expression (Liu et al., 2015b, Liu et al., 2016). For example, berberine mediates inhibition of cartilage degeneration in IL-1β-stimulated rat chondrocytes through downregulation of IL-1β in a Akt-dependent fasion (Zhao et al., 2014). Besides, following deactivation of JNK signaling pathways by berberine, lower levels of TNF-α and IL-1β have been observed in acute pancreatitis disease (Choi et al., 2016).

Likewise, in present work our finding indicates that IL-1β level signtificantly was increased in MCAO group than control animals (2200pikel/um2), indicating a potent detrimental effect of this proinflammatory cytokine in cerebral ischemia. Decreased level of IL-1β was found in treatment group (*p* < 0.05).

On the other hand, berberine can increase the expression of anti-inflammatory cytokines. In work by Kim and coworker berberine not only decreases the expression of iNOS, COX-2, IL-1β, IL-6, and TNF-α, but also enhances IL-10 expression to inhibit lipid peroxidation in inflammatory bowel diseases (Lee et al., 2010). We examined the effects of berberine on anti-inflammatory cytokines.

**Fig. 5:**
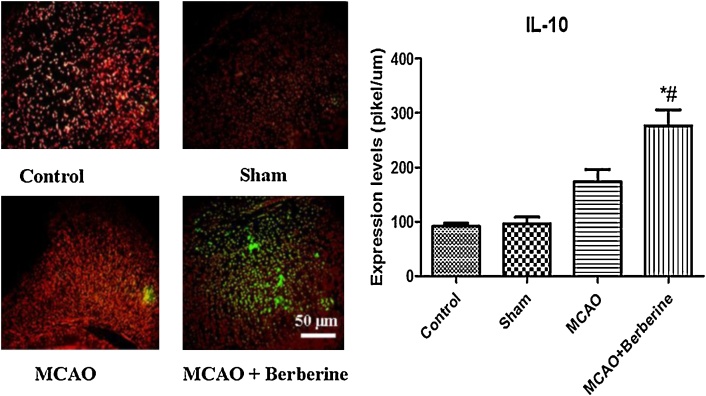
Following MCAO, IL-10 level was increased compared to control and sham groups. A robust increase in IL-10 anti-inflamatory cytokine was observed after treatment by berberine in cortex region (^#^*p* < 0.05 compared with MCAO group) (*p < 0.001 compared to control and sham groups).

We found that focal cerebral ischemia increased IL-10 expression (200pikel/um) compared to control animals (less of 100 pikel/um), whereas berberine dramatically elevated IL-10 levels more than both control and ischemia groups (270 pikel/um), again confirming potent anti-inflammatory effects of berberine (Fig. 5).

## 4 Conclusion

In conclusion, our findings indicate the potent anti-inflamatory effects of berberine on MCAO model in Wistar rats. Our results show that berberine ameliorates infarct volume, brain edema formation, and contributes to recovery of motor function after focal cerebral ischemia via downregulation of pro-inflammatory cytokines and upregulation of anti-inflammatory cytokines.

## Conflict of interest

The authors declare no conflict of interest.

## References

[bib0005] Ajami M., Pazoki-Toroudi H., Amani H., Nabavi S.F., Braidy N., Vacca R.A., Atanasov A.G., Mocan A., Nabavi S.M. (2016). Therapeutic role of sirtuins in neurodegenerative disease and their modulation by polyphenols. Neurosci. Biobehav. Rev..

[bib0010] Chai Y.-S., Yuan Z.-Y., Lei F., Wang Y.-G., Hu J., Du F., Lu X., Jiang J.-F., Xing D.-M., Du L.-J. (2014). Inhibition of retinoblastoma mRNA degradation through Poly (A) involved in the neuroprotective effect of berberine against cerebral ischemia. PLOS ONE.

[bib0015] Chen C.-C., Hung T.-H., Lee C.Y., Wang L.-F., Wu C.-H., Ke C.-H., Chen S.-F. (2014). Berberine protects against neuronal damage via suppression of glia-mediated inflammation in traumatic brain injury. PLOS ONE.

[bib0020] Chen F., Yang Z., Liu Y., Li L., Liang W., Wang X., Zhou W., Yang Y., Hu R.-M. (2008). Berberine inhibits the expression of TNFα, MCP-1, and IL-6 in AcLDL-stimulated macrophages through PPARγ pathway. Endocrine.

[bib0025] Chen W., Wei S., Yu Y., Xue H., Yao F., Zhang M., Xiao J., Hatch G.M., Chen L. (2016). Pretreatment of rats with increased bioavailable berberine attenuates cerebral ischemia-reperfusion injury via down regulation of adenosine-5′ monophosphate kinase activity. Eur. J. Pharmacol..

[bib0030] Chen X., Zhang X., Wang Y., Lei H., Su H., Zeng J., Pei Z., Huang R. (2015). Inhibition of immunoproteasome reduces infarction volume and attenuates inflammatory reaction in a rat model of ischemic stroke. Cell Death Dis..

[bib0035] Choi S.-B., Bae G.-S., Jo I.-J., Wang S., Song H.-J., Park S.-J. (2016). Berberine inhibits inflammatory mediators and attenuates acute pancreatitis through deactivation of JNK signaling pathways. Mol. Immunol..

[bib0040] Feigin V.L., Forouzanfar M.H., Krishnamurthi R., Mensah G.A., Connor M., Bennett D.A., Moran A.E., Sacco R.L., Anderson L., Truelsen T. (2014). Global and regional burden of stroke during 1990–2010: findings from the Global Burden of Disease Study 2010. Lancet.

[bib0045] Guo T., Woo S.-L., Guo X., Li H., Zheng J., Botchlett R., Liu M., Pei Y., Xu H., Cai Y. (2016). Berberine ameliorates hepatic steatosis and suppresses liver and adipose tissue inflammation in mice with diet-induced obesity. Sci. Rep..

[bib0050] Hu J., Chai Y., Wang Y., Kheir M.M., Li H., Yuan Z., Wan H., Xing D., Lei F., Du L. (2012). PI3K p55γ promoter activity enhancement is involved in the anti-apoptotic effect of berberine against cerebral ischemia–reperfusion. Eur. J. Pharmacol..

[bib0055] Jazayeri M., Amani H., Pourfatollah A., Avan A., Ferns G., Pazoki-Toroudi H. (2016). Enhanced detection sensitivity of prostate-specific antigen via PSA-conjugated gold nanoparticles based on localized surface plasmon resonance: GNP-coated anti-PSA/LSPR as a novel approach for the identification of prostate anomalies. Cancer Gene Ther..

[bib0060] Jazayeri M.H., Amani H., Pourfatollah A.A., Pazoki-Toroudi H., Sedighimoghaddam B. (2016). Various methods of gold nanoparticles (GNPs) conjugation to antibodies. Sens. Bio-Sens. Res..

[bib0065] Jeong H.W., Hsu K.C., Lee J.-W., Ham M., Huh J.Y., Shin H.J., Kim W.S., Kim J.B. (2009). Berberine suppresses proinflammatory responses through AMPK activation in macrophages. Am. J. Physiol.-Endocrinol. Metab..

[bib0070] Kim S., Choi J.H., Kim J.B., Nam S.J., Yang J.-H., Kim J.-H., Lee J.E. (2008). Berberine suppresses TNF-α-induced MMP-9 and cell invasion through inhibition of AP-1 activity in MDA-MB-231 human breast cancer cells. Molecules.

[bib0075] Lee C.-H., Chen J.-C., Hsiang C.-Y., Wu S.-L., Wu H.-C., Ho T.-Y. (2007). Berberine suppresses inflammatory agents-induced interleukin-1β and tumor necrosis factor-α productions via the inhibition of IκB degradation in human lung cells. Pharmacol. Res..

[bib0080] Lee I.-A., Hyun Y.-J., Kim D.-H. (2010). Berberine ameliorates TNBS-induced colitis by inhibiting lipid peroxidation, enterobacterial growth and NF-κB activation. Eur. J. Pharmacol..

[bib0085] Li Z., Geng Y.-N., Jiang J.-D., Kong W.-J. (2014). Antioxidant and anti-inflammatory activities of berberine in the treatment of diabetes mellitus. Evid.-Based Complement. Altern. Med..

[bib0090] Liu P., Zhao H., Wang R., Wang P., Tao Z., Gao L., Yan F., Liu X., Yu S., Ji X. (2015). MicroRNA-424 protects against focal cerebral ischemia and reperfusion injury in mice by suppressing oxidative stress. Stroke.

[bib0095] Liu S.-C., Lee H.-P., Hung C.-Y., Tsai C.-H., Li T.-M., Tang C.-H. (2015). Berberine attenuates CCN2-induced IL-1β expression and prevents cartilage degradation in a rat model of osteoarthritis. Toxicol. Appl. Pharmacol..

[bib0100] Liu Y.-F., Wen C.-Y.-Z., Chen Z., Wang Y., Huang Y., Tu S.-H. (2016). Effects of berberine on NLRP3 and IL-1β expressions in monocytic THP-1 cells with monosodium urate crystals-induced inflammation. BioMed Res. Int..

[bib0105] Liu Z., Shen Y., Wu Y., Yang Y., Wu J., Zhou P., Lu X., Guo Z. (2013). An intrinsic therapy of gold nanoparticles in focal cerebral ischemia-reperfusion injury in rats. J. Biomed. Nanotechnol..

[bib0110] Longa E.Z., Weinstein P.R., Carlson S., Cummins R. (1989). Reversible middle cerebral artery occlusion without craniectomy in rats. Stroke.

[bib0115] Lu J.-J., Fu L., Tang Z., Zhang C., Qin L., Wang J., Yu Z., Shi D., Xiao X., Xie F. (2016). Melatonin inhibits AP-2β/hTERT, NF-κB/COX-2 and Akt/ERK and activates caspase/Cyto C signaling to enhance the antitumor activity of berberine in lung cancer cells. Oncotarget.

[bib0120] Manley G.T., Fujimura M., Ma T., Noshita N., Filiz F., Bollen A.W., Chan P., Verkman A. (2000). Aquaporin-4 deletion in mice reduces brain edema after acute water intoxication and ischemic stroke. Nat. Med..

[bib0125] Masada T., Hua Y., Xi G., Yang G.-Y., Hoff J.T., Keep R.F. (2001). Attenuation of intracerebral hemorrhage and thrombin-induced brain edema by overexpression of interleukin-1 receptor antagonist. J. Neurosurg..

[bib0130] Mehrjerdi F.Z., Aboutaleb N., Habibey R., Ajami M., Soleimani M., Arabian M., Niknazar S., Davoodi S.H., Pazoki-Toroudi H. (2013). Increased phosphorylation of mTOR is involved in remote ischemic preconditioning of hippocampus in mice. Brain Res..

[bib0135] Pazoki-Toroudi H., Amani H., Ajami M., Nabavi S.F., Braidy N., Kasi P.D., Nabavi S.M. (2016). Targeting mTOR signaling by polyphenols: a new therapeutic target for ageing. Ageing Res. Rev..

[bib0140] Refaat A., Abdelhamed S., Yagita H., Inoue H., Yokoyama S., Hayakawa Y., Saiki I. (2013). Berberine enhances tumor necrosis factor-related apoptosis-inducing ligand-mediated apoptosis in breast cancer. Oncol. Lett..

[bib0145] Saki K., Eftekhari Z., Naghdi N., Bahmani M. (2016). Berberis vulgaris as an antihypertensive drug; berbamine and oxycontin antihypertensive active ingredients. J. Prevent. Epidemiol..

[bib0150] Shinohara N., Nakamura T., Abe Y., Hifumi T., Kawakita K., Shinomiya A., Tamiya T., Tokuda M., Keep R.F., Yamamoto T. (2016). d-Allose attenuates overexpression of inflammatory cytokines after cerebral ischemia/reperfusion injury in gerbil. J. Stroke Cerebrovasc. Dis..

[bib0155] Singh D.P., Chopra K. (2013). Verapamil augments the neuroprotectant action of berberine in rat model of transient global cerebral ischemia. Eur. J. Pharmacol..

[bib0160] Spatuzza C., Postiglione L., Covelli B., Ricciardone M., Benvenuti C., Mondola P., Belfiore A. (2014). Effects of berberine and red yeast on proinflammatory cytokines IL-6 and TNF-α in peripheral blood mononuclear cells (PBMCs) of human subjects. Front. Pharmacol..

[bib0165] Wang F., Zhao G., Cheng L., Zhou H.-Y., Fu L.-Y., Yao W.-X. (2004). Effects of berberine on potassium currents in acutely isolated CA1 pyramidal neurons of rat hippocampus. Brain Res..

[bib0170] Wang Y., Yi X., Ghanam K., Zhang S., Zhao T., Zhu X. (2014). Berberine decreases cholesterol levels in rats through multiple mechanisms, including inhibition of cholesterol absorption. Metabolism.

[bib0175] Wen S.-Q., Jeyakkumar P., Avula S.R., Zhang L., Zhou C.-H. (2016). Discovery of novel berberine imidazoles as safe antimicrobial agents by down regulating ROS generation. Bioorg. Med. Chem. Lett..

[bib0180] Woo S.-L., Guo T., Guo X., Li H., Zheng J., Botchlett R., Cai Y., Li X., Li Q., Xiao X. (2016). Berberine ameliorates hepatic steatosis and suppresses liver and adipose tissue inflammation in obesity mice independent of AMPK. FASEB J..

[bib0185] Wu J., Shi Y., Liu T. (1994). Protective effects of berberine on cerebral ischemia in mice and rats. Chin. J. Pharmacol. Toxicol..

[bib0190] Yao X., Derugin N., Manley G.T., Verkman A. (2015). Reduced brain edema and infarct volume in aquaporin-4 deficient mice after transient focal cerebral ischemia. Neurosci. Lett..

[bib0195] Ye M., Fu S., Pi R., He F. (2009). Neuropharmacological and pharmacokinetic properties of berberine: a review of recent research. J. Pharm. Pharmacol..

[bib0200] Yu L., Li F., Zhao G., Yang Y., Jin Z., Zhai M., Yu W., Zhao L., Chen W., Duan W. (2015). Protective effect of berberine against myocardial ischemia reperfusion injury: role of Notch1/Hes1-PTEN/Akt signaling. Apoptosis.

[bib0205] Zhang Q., Bian H., Guo L., Zhu H. (2016). Pharmacologic preconditioning with berberine attenuating ischemia-induced apoptosis and promoting autophagy in neuron. Am. J. Transl. Res..

[bib0210] Zhang X., Li H., Hu S., Zhang L., Liu C., Zhu C., Liu R., Li C. (2006). Brain edema after intracerebral hemorrhage in rats: the role of inflammation. Neurol. India.

[bib0215] Zhang X., Zhang X., Wang C., Li Y., Dong L., Cui L., Wang L., Liu Z., Qiao H., Zhu C. (2012). Neuroprotection of early and short-time applying berberine in the acute phase of cerebral ischemia: up-regulated pAkt, pGSK and pCREB, down-regulated NF-κB expression, ameliorated BBB permeability. Brain Res..

[bib0220] Zhao G.-l., Yu L.-m., Gao W.-l., Duan W.-x., Jiang B., Liu X.-d., Zhang B., Liu Z.-h., Zhai M.-e., Jin Z.-x. (2016). Berberine protects rat heart from ischemia/reperfusion injury via activating JAK2/STAT3 signaling and attenuating endoplasmic reticulum stress. Acta Pharmacol. Sin..

[bib0225] Zhao H., Zhang T., Xia C., Shi L., Wang S., Zheng X., Hu T., Zhang B. (2014). Berberine ameliorates cartilage degeneration in interleukin-1β-stimulated rat chondrocytes and in a rat model of osteoarthritis via Akt signalling. J. Cell. Mol. Med..

[bib0230] Zhou X.-Q., Zeng X.-N., Kong H., Sun X.-L. (2008). Neuroprotective effects of berberine on stroke models in vitro and in vivo. Neurosci. Lett..

